# A New Role for Wilms Tumor Protein 1: Differential Activities of + KTS and –KTS Variants to Regulate LHβ Transcription

**DOI:** 10.1371/journal.pone.0116825

**Published:** 2015-01-24

**Authors:** Debalina Bagchi, Josefa Andrade, Margaret A. Shupnik

**Affiliations:** 1 Department of Cell Biology, University of Virginia School of Medicine, Charlottesville, Virginia, United States of America; 2 Department of Medicine, Division of Endocrinology, University of Virginia School of Medicine, Charlottesville, Virginia, United States of America; John Hopkins University School of Medicine, UNITED STATES

## Abstract

Luteinizing hormone (LH) is synthesized and secreted throughout the reproductive cycle from gonadotrope cells in the anterior pituitary, and is required for steroidogenesis and ovulation. LH contains an α-subunit common with FSH, and a unique LHβ subunit that defines biological activity. Basal LHβ transcription is low and stimulated by hypothalamic GnRH, which induces synthesis of early growth response protein-1 (Egr1), and stimulates binding of transcription factors Egr1 and steroidogenic factor-1 (SF1) on the promoter. WT1 (Wilms tumor protein1) is a zinc finger transcription factor with an essential role in urogenital system development, and which regulates several reproductive genes via interactions with SF1 or binding to GC-rich elements such as Egr1 binding sites. We investigated a potential role for WT1 in LHβ transcription in clonal mouse gonadotrope LβT2 cells. WT1 was present in LβT2 and mouse pituitary cells, and protein bound to the endogenous LHβ promoter. Interestingly, mRNAs for WT1(+KTS), which contains a three amino-acid insertion between the 3^rd^ and 4^th^ zinc fingers, and the WT1 (-KTS) variant were both expressed at significant levels. WT1 mRNAs and protein were decreased approximately 50% by GnRH treatment, under conditions where Egr1 mRNA and protein, and LHβ transcription, were stimulated. Decreasing expression of mRNA for WT1 (-KTS) decreased stimulation of LHβ and Egr1 by GnRH, whereas decreasing both WT1 (-KTS) and (+KTS) increased endogenous LHβ transcription, and prevented LHβ but not Egr1 stimulation by GnRH, suggesting differing biological activities for the WT1 isoforms. Overexpression of WT1 showed that WT1(-KTS) enhanced LHβ promoter GnRH stimulation 2-to-3-fold and required the 3’Egr1 site, but WT1(+KTS) repressed both basal and GnRH-stimulated LHβ promoter activity by approximately 70%. Our data suggest that WT1 can modulate LHβ transcription, with differential roles for the two WT1 variants; WT1 (-KTS) enhances and WT1 (+KTS) suppresses transcription.

## Introduction

Gonadotropin hormones secreted from the anterior pituitary control female reproduction, and Luteinizing Hormone (LH) specifically is necessary for ovulation and steroidogenesis [1, 2]. LH consists of two subunits, an alpha subunit shared with FSH, and a unique beta subunit, which is limiting for the intact hormone [3]. Hypothalamic GnRH is a crucial modulator of the gonadotropin subunit genes, and among all the subunits LHβ is most dramatically and precisely regulated by GnRH [4, 5]. The LHβ promoter includes two GnRH responsive regions. The distal region contains two SP1 sites and a CArG box. The proximal GnRH response element, conserved across all mammalian species including humans, consists of two Egr1 (Early Growth Response 1), two SF1 (Steroidogenic Factor 1) binding sites, and a binding site for the homeobox protein Ptx1. Full transcriptional activation requires interactions and synergy between the distal and proximal response elements [6, 7]. Synthesis of the zinc-finger transcription factor Egr1 (early growth response1) occurs rapidly in response to GnRH and is a critical component of increased LHβ transcription [8, 9, 10, 11]. SF1 is a nuclear receptor that regulates the transcription of several genes involved in steroidogenesis and reproduction, including the pituitary gonadotropin subunit genes and the GnRH receptor [12, 13, 14].

In response to GnRH, co-ordinated binding of transcription factors occurs on the LHβ promoter [10, 15]. These proteins in turn may associate with additional stimulatory and suppressive regulatory proteins, including SNURF [15], SRC-1 [16] and DAX-1 [17, 18] that influence the response of reproductive genes to hormonal and physiological challenges. The WT1 protein (Wilms Tumor protein 1) associates with Sp1, SF1 and DAX-1 to exert influence on many reproductive gene promoters including SF1 itself, and is essential for mammalian urogenital development and gonadogenesis prior to sexual differentiation [18, 19, 20, 21]. In addition, WT1 binds directly to DNA at GC-rich motifs similar to those for Egr1 or Sp1 [22, 23]. In spite of these intriguing associations with the transcription factors involved in LHβ gene transcription, the potential role of WT1 in LHβ gene transcription has not previously been examined.

WT1 has a broad range of target genes and can act as either a transcriptional repressor or activator, in a cell and promoter specific manner. For example, WT1 represses transcription of the human PDGF A chain [24], human telomerase reverse transcriptase [25], and proto-oncogenes bcl-2 and c-myc [26] genes, but stimulates the SF1 [18], DAX-1 [27], erythropoietin [28] and amphiregulin [29] genes. The WT1 gene has ten exons that encode a proline-glutamine rich amino terminal involved in protein-protein interactions, and four zinc-finger domains towards the carboxy-terminal end that bind DNA [19, 21]. There are several splice variants of WT1, the most common of which include +KTS and –KTS, variants resulting in the presence or absence of a three amino acid (KTS) insertion between the third and fourth zinc finger near exon 9 [21]. In this paper, we investigated the role of WT1 in LHβ transcription, addressing the individual roles of the WT1 (+KTS) and WT1 (-KTS) variants under basal and GnRH-stimulated conditions. Our data shows WT1 to be a novel regulator of LHβ and that the splice variants differentially regulate LHβ transcription. The +KTS variant represses both basal and GnRH stimulated LHβ transcription whereas the –KTS variant activates GnRH-stimulated LHβ transcription.

## Materials and Methods

### Cell culture, transient transfection and luciferase assay

Experiments were performed using the clonal mouse gonadotrope cell line, LβT2 as previously described [10]. Cells were maintained in Dulbeccos Minimal Essential Medium (DMEM) with 10%FBS (fetal bovine serum) and 1% antibiotic/antimycotic (GIBCO, Grand Island, New York). For experiments, cells were plated in phenol red-free DMEM with 5% charcoal stripped serum and 2%L-glutamine. GnRH (50 nM, Bachem Biosciences Inc, King of Prussia, PA under the name LHRH) was used as indicated. LβT2 cells were plated using DMEM plus 5% charcoal stripped newborn calf serum at the concentration of 500,000 cells per well in 12-well (20 mm diameter) dishes. After 24h, the cells were transfected with a luciferase reporter construct driven by the rat LHβ promoter using Lipofectamine 2000 (Invitrogen; Carlsbad, California). For transfection experiments, 0.33 μg per reaction of the rat LHβ gene promoter from -617 to +44 bp (containing both distal and proximal GnRH responsive elements) or 1μg per reaction of truncated promoter (-245 to +44 region containing only the downstream GnRH response element) fused to luciferase were used. In WT1 overexpression/dose response studies, increasing concentrations of WT1 (splice variants +KTS and –KTS) plasmid DNA up to 0.33 μg were transfected along with the reporter plasmid. Empty vector pCB6+ plasmid at various concentrations was transfected to keep total DNA constant. WT1 expression plasmids were a generous gift from Dr. Nicholas Webster, UCSD [30]. In some studies, the rat LHβ gene promoter from -617 to +44 bp with previously described mutations in either the SF1 sites or the Egr1 sites [6, 31] were used to define the requirement for these DNA regions in WT1 actions. After 48 h of transfection, the cells were treated with or without GnRH for 6 hrs and the cell lysates were collected in 200 μl of 1x passive lysis buffer (Promega, Madison, WI, cell culture lysis reagent). The samples were centrifuged at 13,000 rpm for 1 min and supernatant was collected. Luciferase activity was measured using a Turner TD-20e luminometer (Turner Designs, Mountain View, CA). Total proteins of each sample were measured using the Bradford Protein assay (Bio-Rad dye; Hercules, CA). Luciferase activity was normalized as described [10, 32]. Average and standard error were calculated for 6 samples. Statistical significance was determined using paired student T-test and ANOVA and with differences between treatment groups determined by Bonferroni multiple comparison test [31].

In some experiments, normal mouse pituitaries were used to measure WT1 mRNAs. Female mice were ovariectomized between 2–3 months of age; approximately 10–14 days post-ovariectomy, animals were treated with oil or 300 ng 17β-estradiol for 3 days as previously described [33, 34]. Animals were killed at 9AM and pituitaries collected for RNA purification and mRNA measurement as previously described [31, 32]. For *in vitro* GnRH treatment, pituitary cells from adult mice were treated in culture with 5 nM GnRH; WT1 mRNAs were measured and normalized for GAPDH mRNA [32].

### siRNA delivery and primary transcript assay

To decrease expression of mRNAs and protein, siRNA was delivered into LβT2 cells using nucleofection technology according to the manufacturer’s instructions (Amaxa Corp., Gaithersburg, MD) and as previously described [32]. The LβT2 cells were nucleofected with siGENOME SMARTpool siRNA (0.2nM;Dharmacon RNA Technologies, Lafayette, CO) directed against mouse WT1 or a non-targeting negative control siRNA (0.2nM, siCON #1; Dharmacon), in Solution T and using Program A-020. Each reaction contained 5 × 10^6^ cells and was divided between three wells in a 35-mm plate containing 2 ml 5% SNCS. Cell lysates were collected after 72 h for primary transcript assay for LHβ, RT-PCR to detect WT1 mRNA isoforms, and immunoblotting to confirm WT1 knockdown. Experiments were performed in triplicate three times. Protein levels were analyzed on immunoblots.

To measure specific mRNAs, RNA was isolated from the cell lysates using the QIAGEN (Valencia, CA) RNeasy kit and was briefly treated with DNase (Roche, Indianapolis, IN) to remove DNA contamination. Total RNA was subjected to reverse transcription of the mRNA using the iScript cDNA synthesis kit (Bio-Rad). Quantitative real-time PCR was performed using cDNA as template, as described [31, 32]. Primers were designed against the first intron/exon border of the mouse LHβ gene to detect unspliced mRNA primary transcript (PT) (forward primer sequence, 5’-AGAGGCTCCAGGTAAGATGGTA-3’; reverse primer sequence, 5’-CCACTCAGTATAATACAGAAAC-3’). Egr1 primary transcript mRNA was measured as previously described [10, 32]. Both Egr1 and LHβ primary transcript mRNAs were normalized to GAPDH mRNA levels. Samples without the reverse transcriptase enzyme during cDNA synthesis were used as negative controls. In results, five representative experiments among seven experiments are shown. To measure expression of WT1 (splice variants +KTS and –KTS) mRNA, cells were treated with vehicle or 50 nM GnRH for 90 min, RNA was isolated and quantitative PCR was performed. The WT1 primers used were: forward primer sequence, 5–CATCTGAAACCAGTGAGAAACG–3; reverse primer sequence for -KTS, 5–CTCATACAGGTGAAAAGCCCTT–3, reverse primer sequence for +KTS, 5–CTCATACAGGTAAAACAAGTGAAAAGCCCTT–3. All mRNAs were normalized to GAPDH mRNA levels. Averages and SEM were calculated from PCR replicates.

### Western Blot

For Western blot analysis, 2 × 10^6^ cells per well were plated in 6-well 35 mm dishes. After 24h, cells were treated with 50 nM GnRH and collected every 30 min for 3.5 h. Cells were lysed and collected using 2x gel loading buffer [100 mM Tris-HCL (pH 6.8), 4% SDS, 20% glycerol] plus protease inhibitors as described by Andrade et al. [32], and the protein concentration was measured using the Pierce (Rockford, IL) BCA Kit. Cell lysates were heated for 5 min at 95 C and equal amounts of proteins of each sample were separated by 10% SDS-PAGE using 140V constant voltage for approximately 2 h. Proteins were transferred to a nitrocellulose membrane using 35V constant voltage for 3 h. Membranes were then blocked using 10% non-fat dry milk in Tris-buffered saline plus 1% Tween 20 (TBST) for 1h in room temperature. Membranes were then incubated with a WT1 primary antibody (C-19:SC-192 Santa Cruz Biotechnologies, Santa Cruz CA) overnight (1:500) or Egr1 primary antibody (Cell Signaling Technology) overnight (1:1000) at 4C followed by three 5 min washes with TBST and another incubation with secondary antibody, horseradish peroxidase-conjugated donkey anti-rabbit Fab fragment IgG (1:5000;GE HealthCare; Piscataway, New Jersey) for 2h. Relative levels of proteins were detected with ECL, SuperSignal West Pico Chemiluminescent Substrate (Pierce, Rockford, IL) and X-ray film autoradiography. Monoclonal anti-β-actin primary antibody (Sigma) was used as a loading control to re-probe the membrane. Band intensity was measured by densitometry and normalized against β-actin in the same samples.

### Chromatin Immunoprecipitation

Assays were performed as previously described [10, 31]. To measure WT1 promoter occupancy under basal conditions (without GnRH treatment), LβT2 cells were pre-treated with 2.5 μM of α-amanitin for 1h followed by thorough washing with PBS and incubation with phenol red-free DMEM with 5% charcoal stripped serum and 2%L-glutamine for 20 min. For GnRH studies, LβT2 cells were first pre-treated with 2.5 μM of α-amanitin for 1h to synchronize protein occupancy on the promoter, washed, then incubated in fresh media with or without 50 nM GnRH and two plates of cells collected every 10 min for 2h. Chromatin immunoprecipitation (ChIP) assays were performed by cross-linking the chromatin from each collected sample with 1% formaldehyde for 10 min; the reaction was stopped by addition of 1.25 M glycine and cells were collected in cold PBS plus protease inhibitors [10, 31]. Cross-linked chromatin was sonicated to approximate lengths of 1000 bp, using a cup horn sonicator (Misonix, Farmingdale, NY). Whole-cell extract was diluted with ChIP sonication buffer plus protease inhibitors, divided into aliquots from each sample at each time point to measure input chromatin DNA, and for immunoprecipiation with specific antibodies. Aliquots were incubated with and without primary antibody overnight at 4 C. Antibodies for WT1 (SC-192), Egr1 (SC-189X), or RNA polymerase II (CTD4H8) were obtained from Santa Cruz Biotechnology (WT1 and Egr1), or Millipore, respectively. Protein G PLUS agarose beads (SC-2002; Santa Cruz Biotechnology) were then added for 2 h at 4C to precipitate the antibody bound chromatin. Agarose beads were washed with sonication buffer and Tris-EDTA buffer. DNA-protein complex was released with elution buffer and cross-links were reversed by incubation with NaCl at 65 C overnight. DNA was purified using the QIAGEN PCR purification kit and promoter occupancy was measured with quantitative real-time PCR (iCycler; Bio-Rad) as described [10]. The primers used were located at—102bp (Forward 5–CTGTGTCTCGCCCCCAAAGAGATTA–3) and -1bp (reverse 5–CCTGGCTTTATACCTGCGGGGTT–3) to detect the LHβ promoter. Each individual sample was corrected for background and normalized for total chromatin input.

### Ethics Statement

LβT2 cells were obtained from Dr. Pamela Mellon, University of California, San Diego, who originally developed the line and described their use [35]. Cells were obtained in 1999, at at passage 14, and immediately split into multiple flasks to freeze and keep as close to the original founder line as possible; each thaw is accompanied by a similar round of propagation and freezing and cells are not continuously propagated. Recent studies were performed with cells thawed from passages between 19–21. Cells are thawed in Dulbeccos Minimal Essential Medium (DMEM) with 10%FBS (fetal bovine serum) and 1% antibiotic/antimycotic (GIBCO, Grand Island, New York), and have been tested periodically to ensure no micoplasma contamination.

## Results

### WT1 is expressed in LβT2 cells and associates with the LHβ promoter

**Fig 1:**
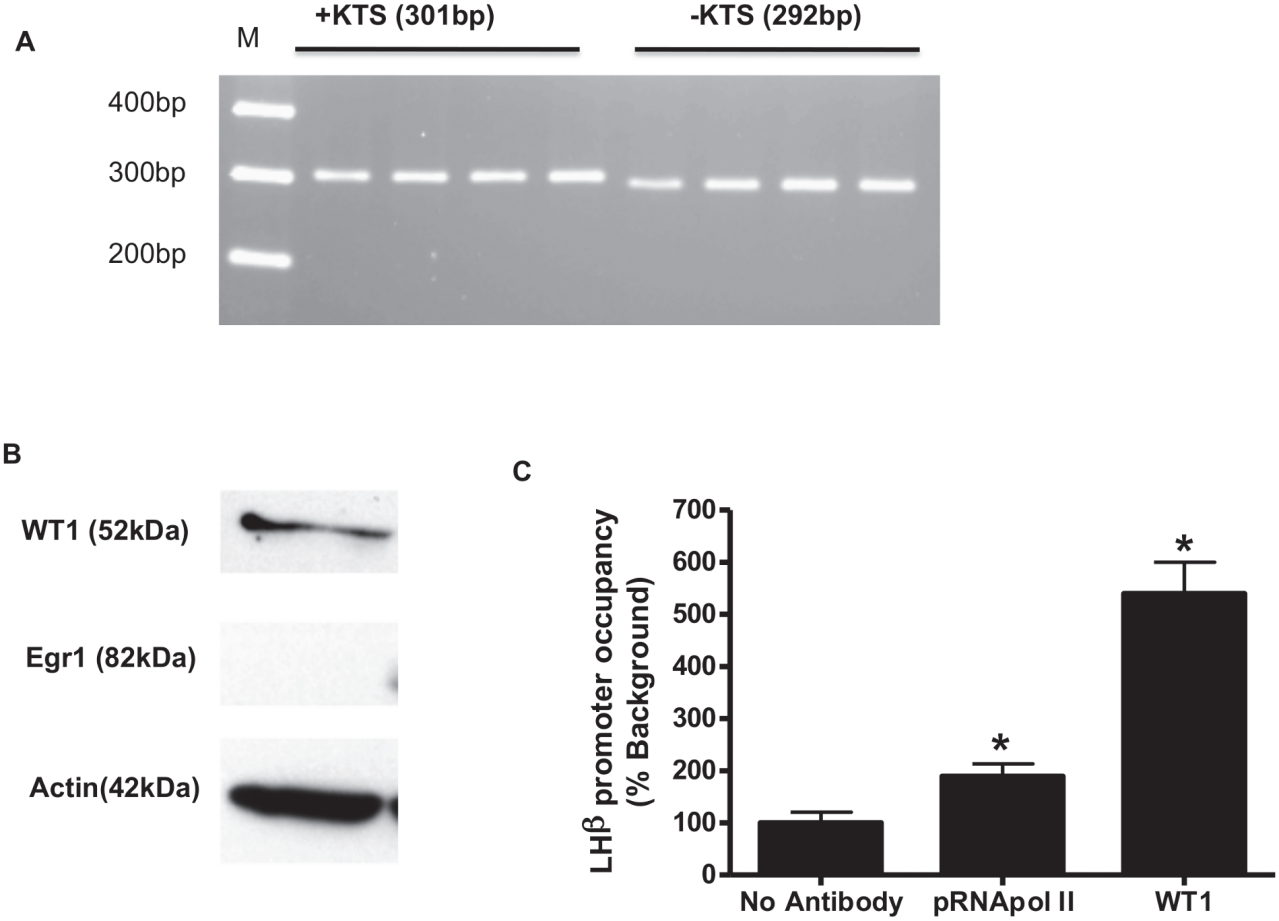
WT1 (-KTS and +KTS) expression and chromatin association in LβT2 cells. A. WT1 mRNA (+KTS and –KTS) splice variant PCR products expressed in LβT2 cells. Whole cell RNA was extracted from LβT2 cells, reverse transcribed to cDNA and quantified by real-time PCR, using specific primers to detect +KTS and –KTS splice variants, then displayed on a 1% agarose gel. Bands corresponding to the products for +KTS (301bp) and –KTS (292 bp) WT1 were detected in 4 independent samples of mRNA. B. WT1 and Egr1 protein expression in LβT2 cells. Cell proteins (30μg) were separated on 10% polyacrylamide-SDS gels, then analyzed by immunoblotting with specific antibodies for WT1, Egr1 and β-actin. Specific proteins were detected in the same samples of untreated LβT2 cells. C. Chromatin association of WT1 with the endogenous LHβ promoter in LβT2 cells. The association of WT1 and RNA Polymerase II with the LHβ promoter in untreated LβT2 cells was measured by Chromatin immunoprecipitation assays with antibodies against WT1 and phosphorylated RNApol II, as well as control (no Antibody). LHβ promoter occupancy was measured by quantitative real time PCR using primers specific for the LHβ promoter, and normalized for chromatin input in each sample. In this study, background binding (no Antibody) was set at 100% and association of RNA Polymerase II and WT1 are expressed relative to background values. Association was measured in 3 independent experiments with duplicate samples.

**Fig 2:**
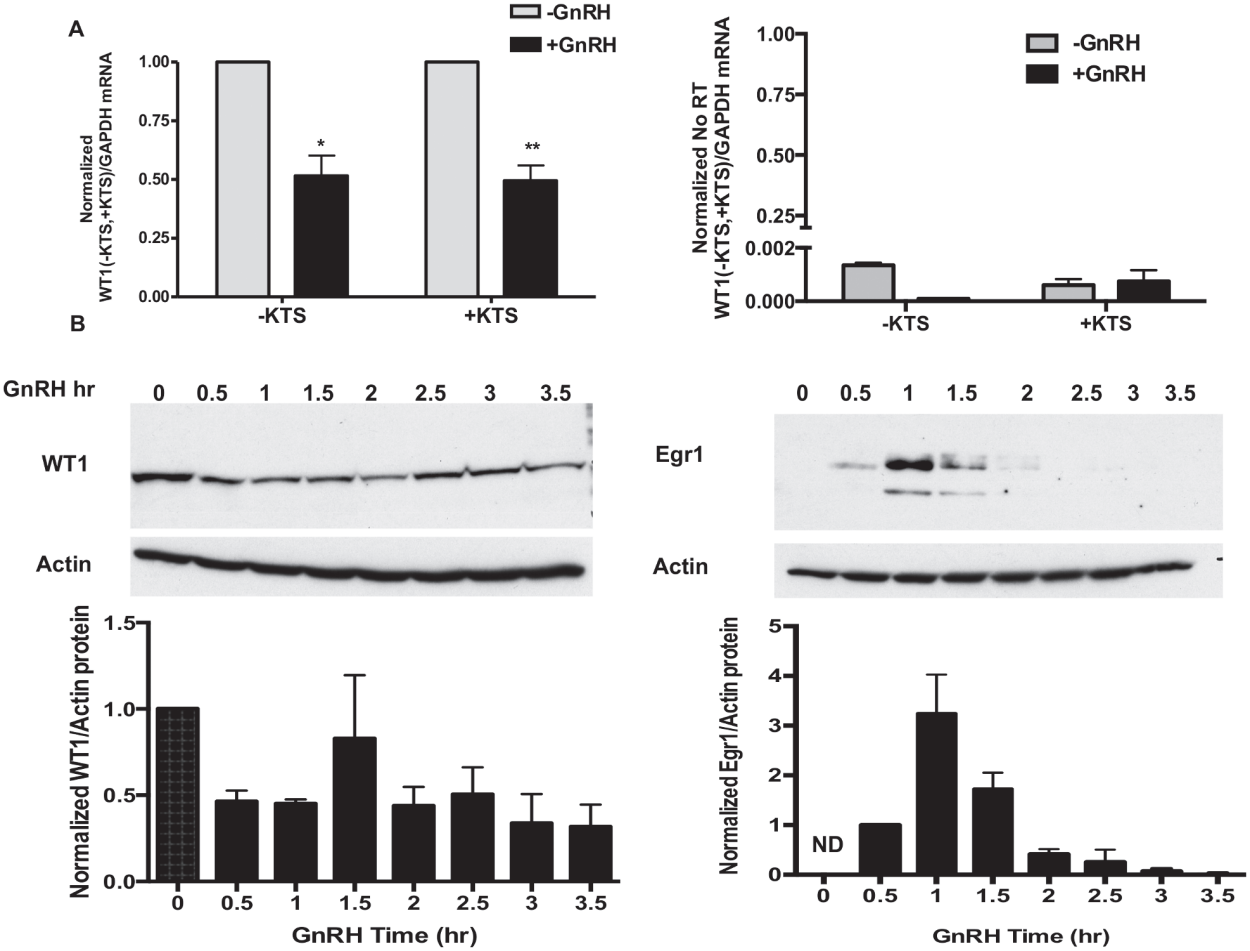
WT1 mRNA and protein levels are decreased by GnRH. A. WT1 (-KTS) and (+ KTS) mRNA variant levels in LβT2 cells treated with 50nM GnRH for 90min. RNA was extracted and mRNAs levels measured by RT-PCR and normalized against GAPDH mRNA. Values for WT1 mRNAs are shown for both Reverse Transcriptase (RT) and no RT (negative control) conditions. Note that for no RT, the Y axis is interrupted and expanded to show the low values. Data is the mean ± SEM from 5–7 experiments. * = P<0.05, -GnRH vs +GnRH; ** = P<0.001, -GnRH vs +GnRH B. WT1 and Egr1 protein levels in LβT2 cells treated with 50nM GnRH for 0 to 3.5 h. The experiment was performed three times. Upper panel: Cells were lysed after GnRH treatment and proteins (30μg) were separated by 10% SDS-PAGE, then detected with antibodies against WT1 or Egr1. Immunoblotting for β-actin was performed on the same blots as WT1 and Egr1, and used for normalization of these proteins quantified by densitometry analysis. A representative blot is shown. Lower panel: Quantification of protein bands was performed with densitometry, and normalized protein levels are shown from combined experiments. Bands for Egr1 proteins were not detected (ND) in any blot at time zero without GnRH.

To determine if the common WT1 variants WT1 (-KTS) and WT1(+KTS) are expressed in LβT2 cells, we first measured WT1 mRNAs by RT-PCR, using specific primers to detect the splice variants. The amplified products, separated on a 1% agarose gel, (Fig. 1A) show bands that correspond to expected sizes for both +KTS (301 bp) and –KTS (292 bp) products; no PCR product was detected without reverse transcriptase either on a gel (not shown) or by incorporation of Syber Green into PCR product (Fig. 2). WT1 protein is also expressed under basal (without GnRH) conditions in LβT2 cells, whereas Egr1, a zinc-finger transcription factor proven to associate with the LHβ promoter, is not expressed at detectable levels under the same conditions (Fig. 1B). Only one specific protein band for WT1 was observed, as expected, as the three amino acid difference cannot be detected by electrophoresis. To determine if WT1 could bind to the endogenous LHβ promoter, chromatin immunoprecipitation assays were performed in untreated LβT2 cells. Under basal conditions (Fig. 1C), both WT1 and phosphorylated RNA Polymerase II associate with the promoter, suggesting that WT1 may play a role in regulating transcription.

### GnRH decreases WT1 mRNA and Protein

The most critical regulatory pathway for LHβ is through GnRH, which induces synthesis of the early response gene and transcription factor Egr1; Egr1 then binds to GC-rich DNA motifs similar to those for Sp1 and WT1 and stimulates transcription [10, 36, 37]. To investigate if WT1 is also regulated by GnRH, LβT2 cells were treated with or without GnRH for 90 min and WT1 (+KTS, -KTS) mRNA levels and WT1 protein levels were measured. Fig. 2 shows that GnRH actually reduces the mRNA levels of both the WT1 splice variants by approximately 50%. Over 10–12 experiments, the relative amounts of +/-KTS variants that were expressed varied somewhat between experiments, as did the degree of GnRH suppression. However, overall the two mRNA variants are both expressed under basal conditions and regulated by GnRH, on average, to the same extent.

We also compared GnRH effects on the protein expression levels of both WT1 and Egr1 in the same experiment. LβT2 cells were treated with GnRH over a period of 0–3.5 h. WT1 or Egr1 proteins were measured by immunoblotting, then normalized to β-actin levels and quantified by densitometric analysis. Fig. 2 shows that WT1 is easily detected in cells without GnRH stimulation, when Egr1 is not expressed. In the presence of GnRH, WT1 protein levels are decreased by approximately 50%, within 30–60 min, but is maintained at this level between 3–3.5h. In contrast, Egr1 protein is transiently and robustly stimulated within 30 min of GnRH treatment, with highest expression levels by 1 h of GnRH. Thus, GnRH differentially regulates WT1 and Egr1, by suppressing WT1 and stimulating Egr1 expression.

### GnRH stimulates WT1 and Egr1 association with the LHβ promoter

**Fig 3:**
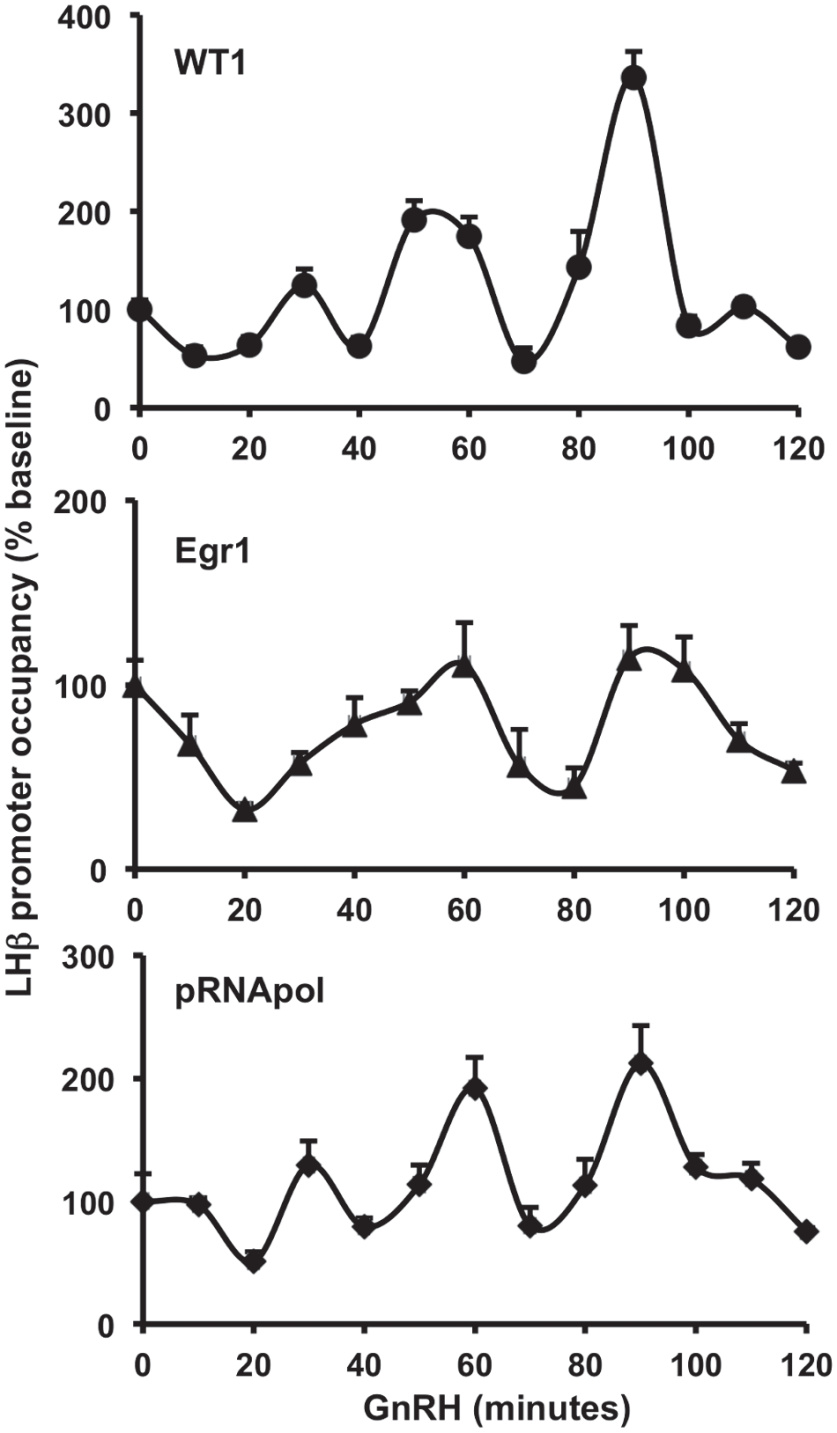
WT1 and Egr1 occupancy of the endogenous LHβ promoter in response to GnRH. LβT2 cells were incubated with or without 50 nM GnRH and collected every 10 min for 120 min. ChIP assays were performed using antibody against WT1, Egr1 and phosphorylated pRNA Pol II. LHβ promoter occupancy was measured by quantitative real time PCR using primers for the LHβ promoter. The experiment was performed three times with duplicate samples and replicate PCR measurements in each sample. Data are presented as the mean + SEM and expressed as LHβ promoter occupancy relative to basal (no GnRH at time zero) binding.

GnRH stimulates the binding of transcription factors and co-regulatory proteins on the LHβ promoter [10]. Since GnRH regulates the mRNA and protein expression levels of Egr1 and WT1, we investigated how GnRH might regulate WT1 association with the LHβ promoter by performing chromatin immunoprecipitation assays in the presence of GnRH (Fig. 3). In the presence of GnRH, transcription factors and RNA Pol II associate with the promoter in a rhythmic fashion, with an interval time of approximately 30 min [10]; both WT1 and pRNA Pol II show this pattern of association. Egr1 is recruited to the promoter with a similar pattern, but with a somewhat delayed association. Maximal Egr1 association required more than 30 min, reaching highest values at 60 min, correlating with the time needed for GnRH-stimulated synthesis, between 30 and 60 min (as in Fig. 2). Thus, WT1 may modulate LHβ transcription at times when Egr1 is present or absent on the promoter.

### Reduced levels of endogenous WT1 influence basal and GnRH-stimulated endogenous LHβ gene transcription

**Fig 4:**
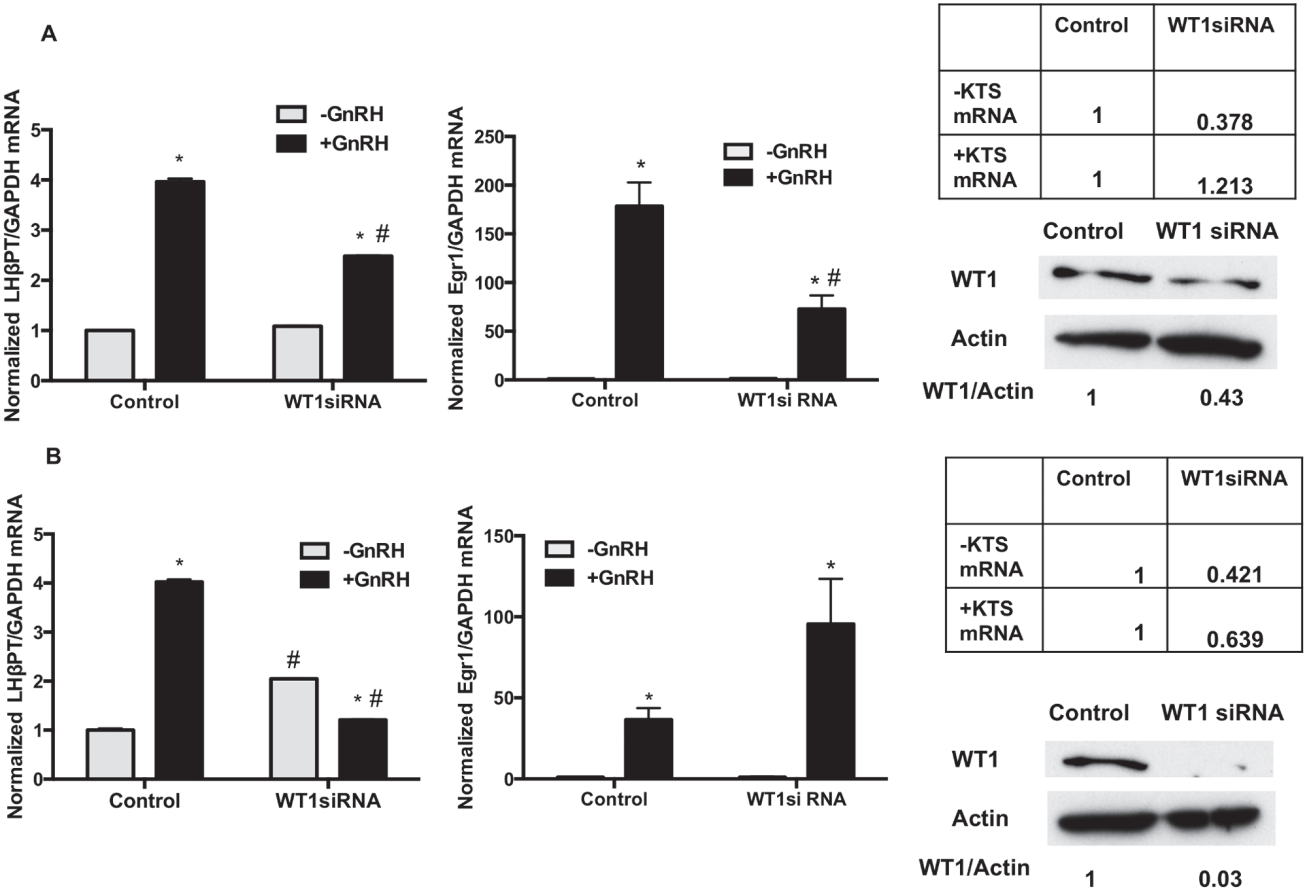
WT1 siRNA increases the basal and decreases GnRH stimulated expression of endogenous LHβ primary transcript. LβT2 cells were transfected with siRNA against WT1 and a non-targeting siRNA as a control. After 72 h of incubation, cells were treated with or without GnRH for 1.5 h, followed by cell lysate collection, RNA extraction and western blot analysis (30 μg protein) on 10% PAGE-SDS gels. WT1 protein was detected by immunoblotting with specific antibodies for WT1, and normalized for β-actin on the same blot. A. Expression of the endogenous LHβ primary transcript, Egr1 primary transcript mRNA, and WT1 protein under conditions where the WT1 (-KTS) variant mRNA was reduced. B. Expression of the endogenous LHβ primary transcript, Egr1 primary transcript mRNA, and WT1 protein under conditions where both the WT1 (-KTS) and WT1 (+KTS) variant mRNAs were reduced. For each condition, the experiment was performed twice with 5 replicates. LHβ and Egr1 primary transcript mRNAs were normalized for GAPDH mRNA in the same sample. Control samples contained non-targeting siRNA. * p<.05 -GnRH vs +GnRH, in either Control siRNA or WT1 siRNA treatments. Values are mean ± SEM for 5 replicates. # = p<.05 -GnRH Control siRNA vs –GnRH WT1 siRNA, or p<.05+GnRH Control siRNA vs +GnRH WT1 siRNA.

To further investigate a potential role for WT1 on endogenous LHβ gene transcription, we decreased endogenous WT1 expression via targeted knock-down by siRNA. A non-targeting siRNA was used in parallel as a control. After 72 h of siRNA treatment, cells were incubated with GnRH for 90 min, and LHβ primary transcript mRNA was measured. As shown in Fig. 4A, knock-down of WT1(-KTS) mRNA alone reduced endogenous WT1 protein by approximately 50%. Under these conditions, basal LHβ mRNA transcription was not significantly affected, but GnRH-stimulated transcription was suppressed by approximately 50%. When both mRNA isoforms for WT1 were decreased, with endogenous WT1 protein decreased by >90% (Fig. 4B), basal LHβ transcription was significantly increased (approximately 2-fold) compared to siControl, and GnRH treatment resulted in lower LHβ primary transcript levels compared to siControl GnRH, or siWT1 cells treated with vehicle. Because WT1 is a transcriptional regulator, and because Egr1 expression is important for LHβ transcription, we also tested the effects of WT1 variant knock-down on the basal and GnRH-stimulated expression of Egr1 mRNA. Interestingly, knock-down of WT1(-KTS) alone reduced GnRH stimulation of Egr1 mRNA by approximately 60%, similar to the suppression of GnRH-stimulated LHβ transcription. In contrast, knock-down of both WT1 isoforms does not suppress Egr1 expression, and GnRH stimulation of Egr1 occurs even though LHβ transcription is not stimulated by GnRH. These data suggest that there may be different, and perhaps even opposing, biological roles for the two WT1 variants on LHβ transcription.

### WT1 +KTS and WT1-KTS variants differentially regulate LHβ promoter activity

**Fig 5:**
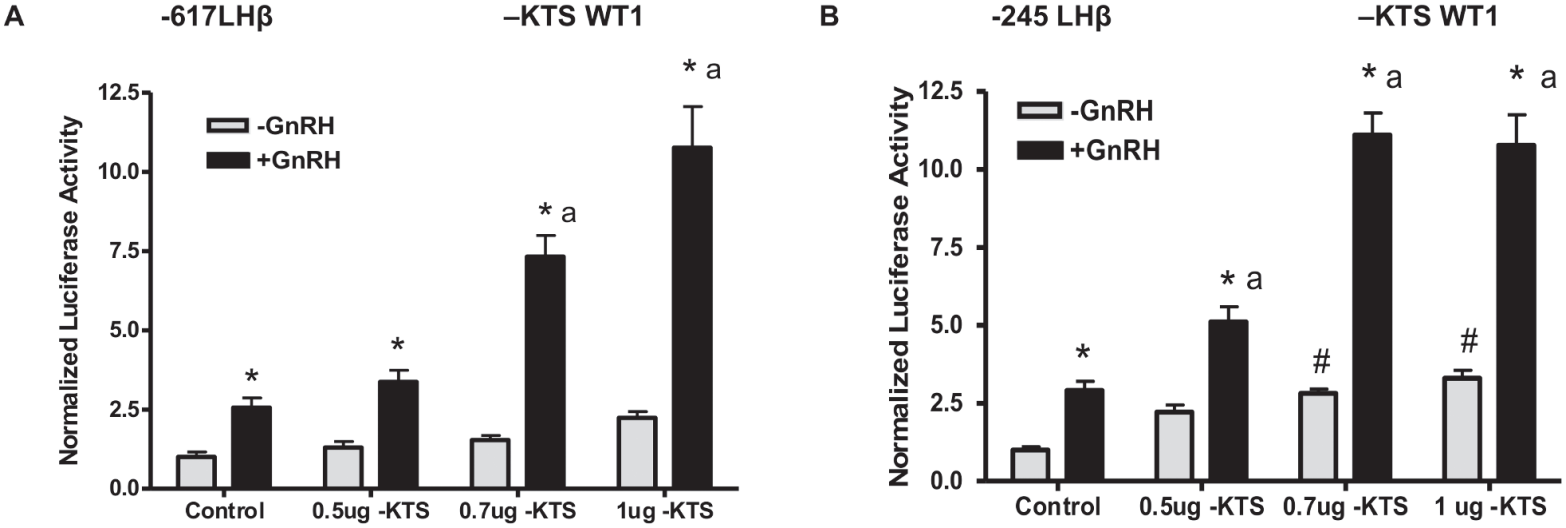
WT1 (-KTS) enhances GnRH-stimulated LHβ promoter activity. LβT2 cells were transfected with either A: A luciferase reporter construct driven by the rat LHβ promoter (-617 to +41 bp) including both distal and proximal GnRH responsive promoter regions, or B: A luciferase reporter construct driven by the rat LHβ promoter (-245 to +44 bp), including only the proximal GnRH response region of the promoter. Constructs were cotransfected with or without 0.5, 0.7, or 1 μg of WT1(-KTS) plasmid, or control plasmid to normalize total DNA. After 48 h post-transfection, cells were treated with 50nM GnRH for 6hrs and collected in lysis buffer. Luciferase activity was measured, and data expressed as average + SE for 6 samples; the experiment was performed 3 times. Statistical significance was determined using ANOVA (confidence interval determined by the Bonferroni multiple comparison test). * p<.05 -GnRH vs +GnRH, # p<.05 control, -GnRH vs –GnRH+WT1, a p<.05 control, +GnRH vs +GnRH +WT1.

**Fig 6:**
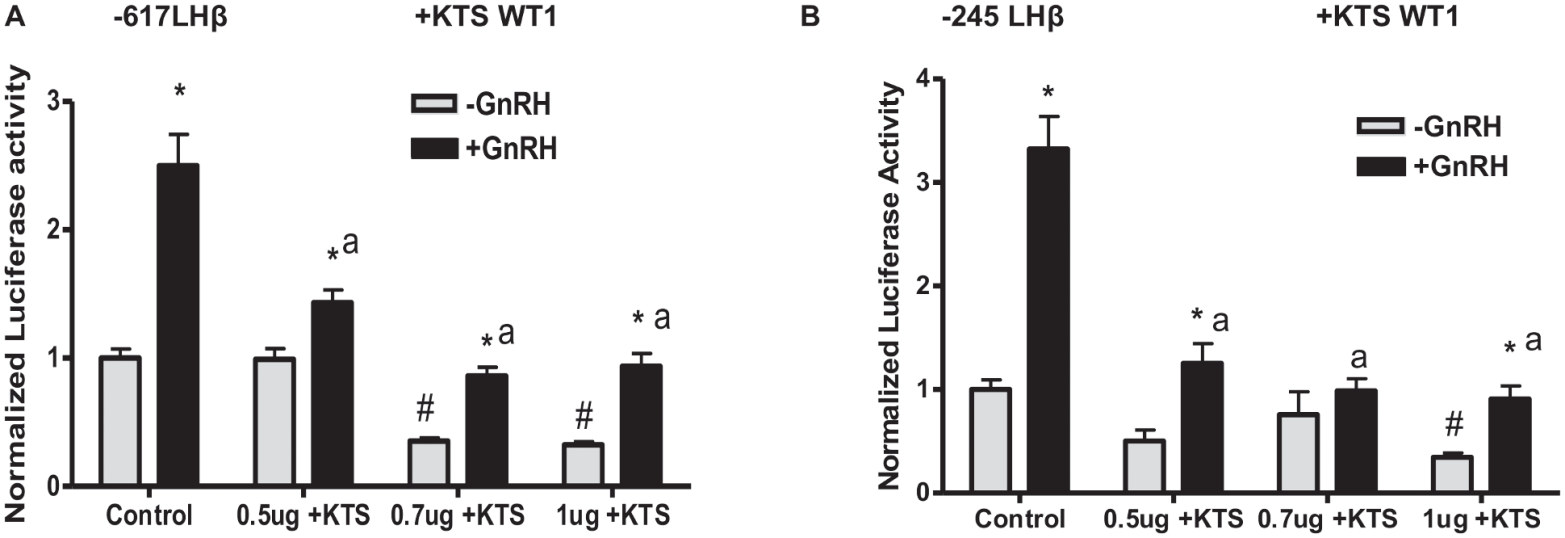
WT1 (+KTS) decreases basal and GnRH-stimulated LHβ promoter activity. LβT2 cells were transfected with either **A)** A luciferase reporter construct driven by the rat LHβ promoter (-617 to +41 bp) including both distal and proximal GnRH responsive promoter regions, or **B)** A luciferase reporter construct driven by the rat LHβ promoter (-245 to +44 bp), including only the proximal GnRH response region of the promoter. Constructs were cotransfected with or without 0.5, 0.7,1 μg of WT1 (+KTS) plasmid or control plasmid to normalize DNA. At 48 h post-transfection, cells were treated with 50nM GnRH for 6 h and collected in lysis buffer. Luciferase activity was measured, and data expressed as average ± SE for 6 samples; the experiment was performed 3 times each. Statistical significance was determined using ANOVA (confidence interval determined by the Bonferroni multiple comparison test). * p<.05 -GnRH vs +GnRH, # = p<.05 control, -GnRH vs –GnRH+WT1, a = p<.05 control, +GnRH vs +GnRH +WT1.

To directly test the role of the WT1 +KTS and –KTS splice variants in LHβ transcription, basal and GnRH-stimulated activity of the LHβ promoter (-617 to +44 bp) was measured in the absence or presence of increasing amounts of WT1(+KTS) or WT1(–KTS) expression vectors. WT1(-KTS) overexpression significantly increased the GnRH stimulation of LHβ promoter activity by approximately 3- to 4-fold at the highest concentrations (Fig. 5A). The -617LHβ promoter contains two regions that might be influenced by WT1, including the distal enhancer that contains Sp1 binding sites, and the proximal enhancer containing Egr1 binding sites; both are required for effective GnRH-stimulated transcription to occur [6, 7]. We thus assessed whether WT1 was able to influence LHβ transcription through the proximal region by testing the LHβ luciferase (-245 to +44 bp) construct containing only proximal GnRH response elements. As shown in Fig. 5B, WT1(-KTS) overexpression significantly increased the basal (up to 3-fold) and GnRH stimulated-LHβ promoter activity of the shorter construct up to 4-fold. In contrast, WT1(+KTS) overexpression significantly decreased the basal and GnRH-stimulated LHβ promoter activity of both the -617 LHβ (Fig. 6A) and the shorter -245 bp constructs (Fig. 6B), by up to 70% at the highest WT1 concentrations.

**Fig 7:**
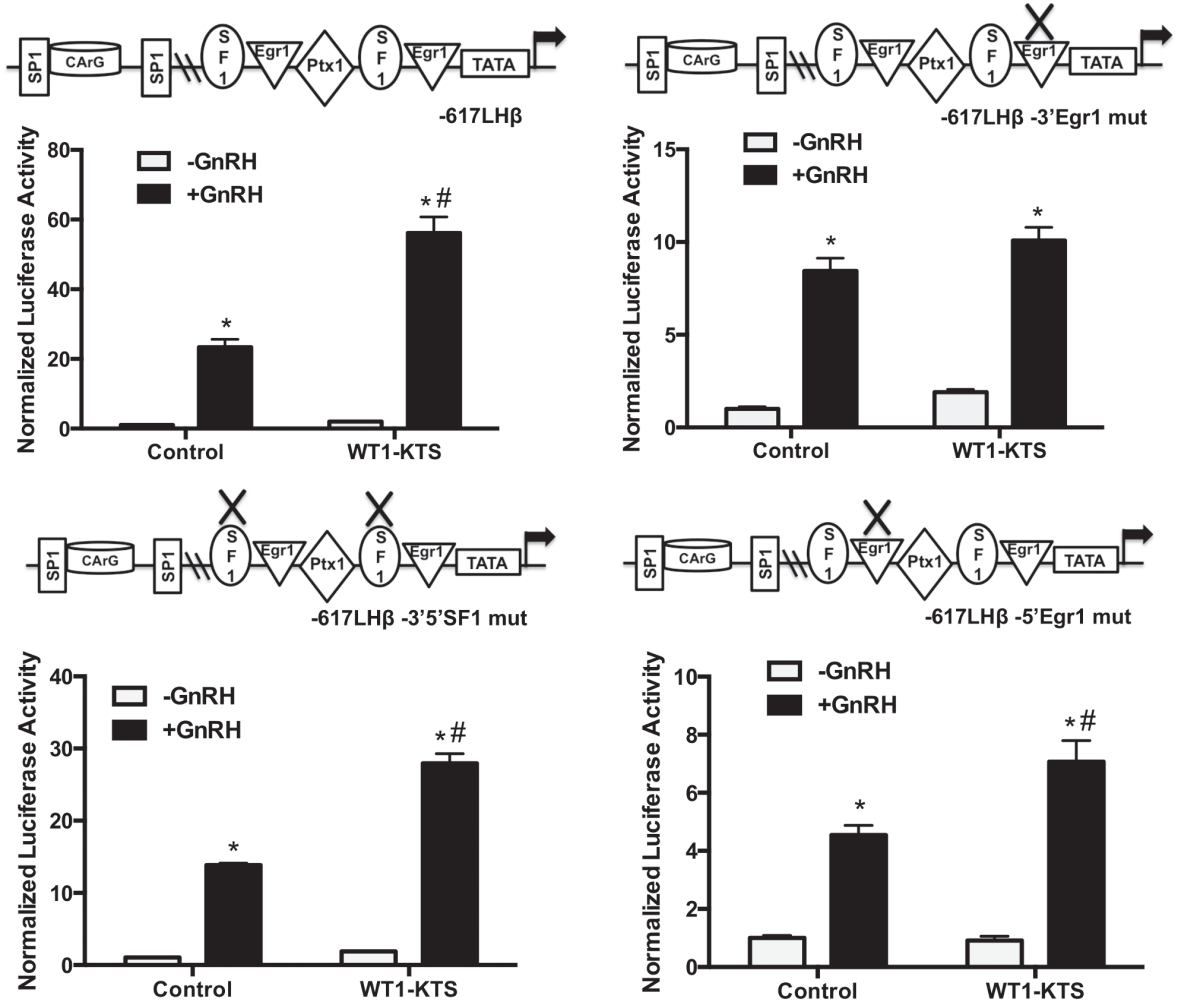
Differential responses of LHβ promoter mutants to WT(-KTS). LβT2 cells were transfected with luciferase constructs containing either the wild type (-617 to +44bp) LHβ promoter, or the same construct mutated at both SF1 sites, or the individual 5’Egr1 or 3’Egr1 sites. Constructs were cotransfected with or without 1μg of WT1(-KTS) plasmid or control plasmid to normalize DNA. At 48 h post-transfection, cells were treated with 50nM GnRH for 6 h and collected in lysis buffer. Luciferase activity was measured, and data expressed as average ± SE for 6 samples; the experiment was performed 3 times each. Statistical significance was determined using ANOVA (confidence interval determined by the Bonferroni multiple comparison test). * p<.05 -GnRH vs +GnRH, # = p<.05 control, -GnRH vs –GnRH+WT1, a = p<.05 control, +GnRH vs +GnRH +WT1.

**Fig 8:**
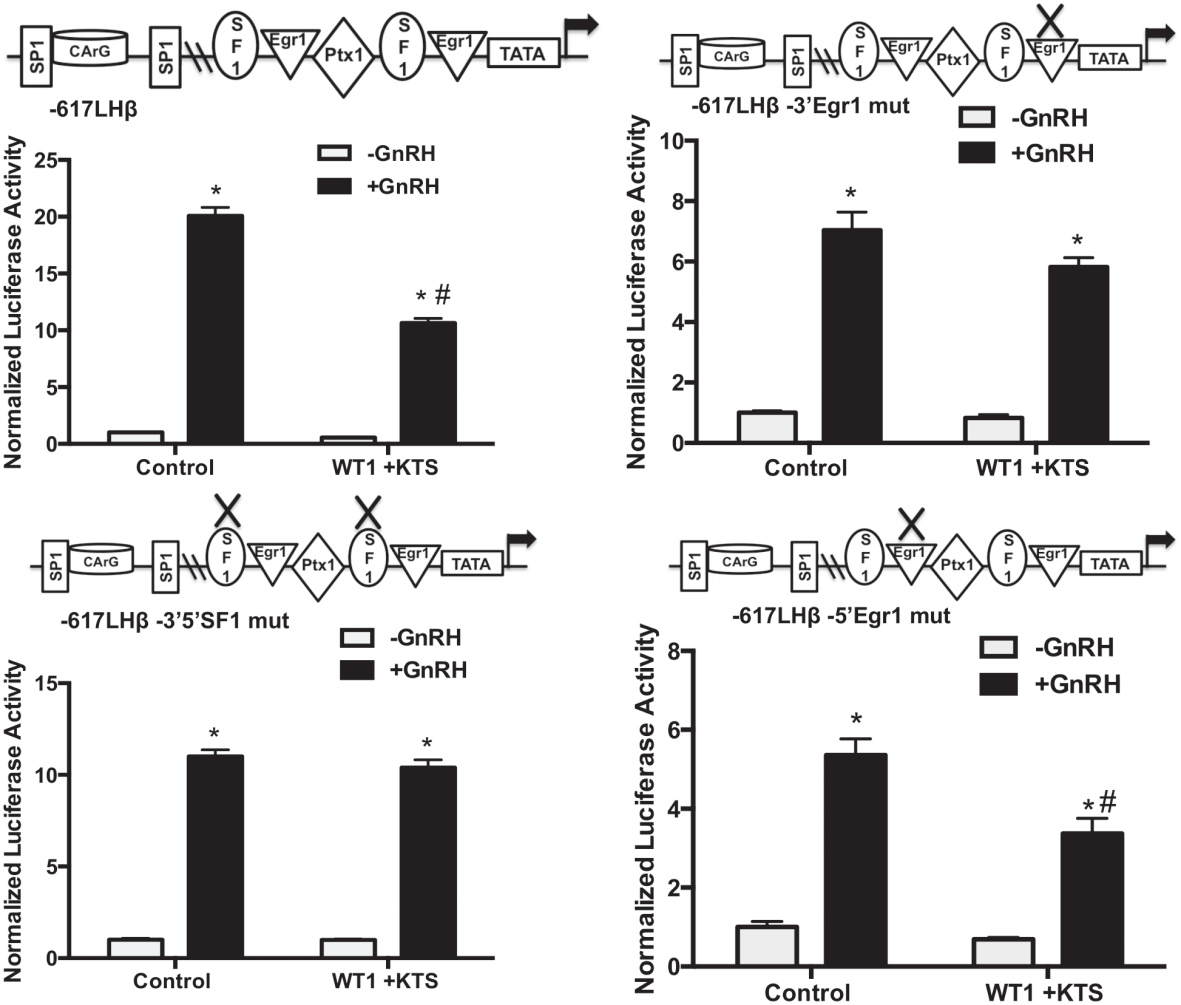
Differential responses of LHβ promoter mutants to WT(+KTS). LHβ promoter mutants were transfected into LβT2 cells with or without 1μg of WT1 (+KTS) plasmid or control plasmid to normalize DNA. Data were analyzed and expressed as in Fig. 7.

Our overexpression data suggests that WT1 splice variants +KTS and –KTS could differentially regulate LHβ transcription, with WT1(–KTS) being a positive regulator and WT1(+KTS) being a negative regulator. In both cases, the proximal promoter containing the Egr1 sites is sufficient to impart WT1 regulation. The proximal GnRH-responsive region also contains binding sites for Ptx1 and SF1; SF1 and Egr1 bind to adjacent sites and all three proteins can form complexes that play an important role in basal and stimulated LHβ transcription [9]. Thus, protein-protein interactions on DNA at Egr1 or SF1 sites may also be important for WT1 effects. To directly test if the WT1 variants act through the same or different DNA elements, LHβ promoter (-617 to +44bp) luciferase constructs containing mutations specifically in both 5’- and 3’-SF1 sites, or individual 3’- or 5-’Egr1 sites, were tested for the response to WT1 variant overexpression in the presence or absence of GnRH. As seen in Fig. 7, mutation of the 3’-Egr1 site, but not the SF1 sites, in the LHβ promoter abrogated the effects of the WT1(-KTS) variant. In contrast, mutation of the SF1 sites or the 3’-Egr1 site eliminated the effects of the WT1(+KTS) variant on the LHβ promoter (Fig. 8). Thus, WT1(-KTS) appears to mediate its effects exclusively through the Egr1 site, whereas the WT1(+KTS) variant requires both Egr1 sites and SF1 sites for its biological effects.

### Expression and regulation of WT1 variants in normal mouse pituitary

**Fig 9:**
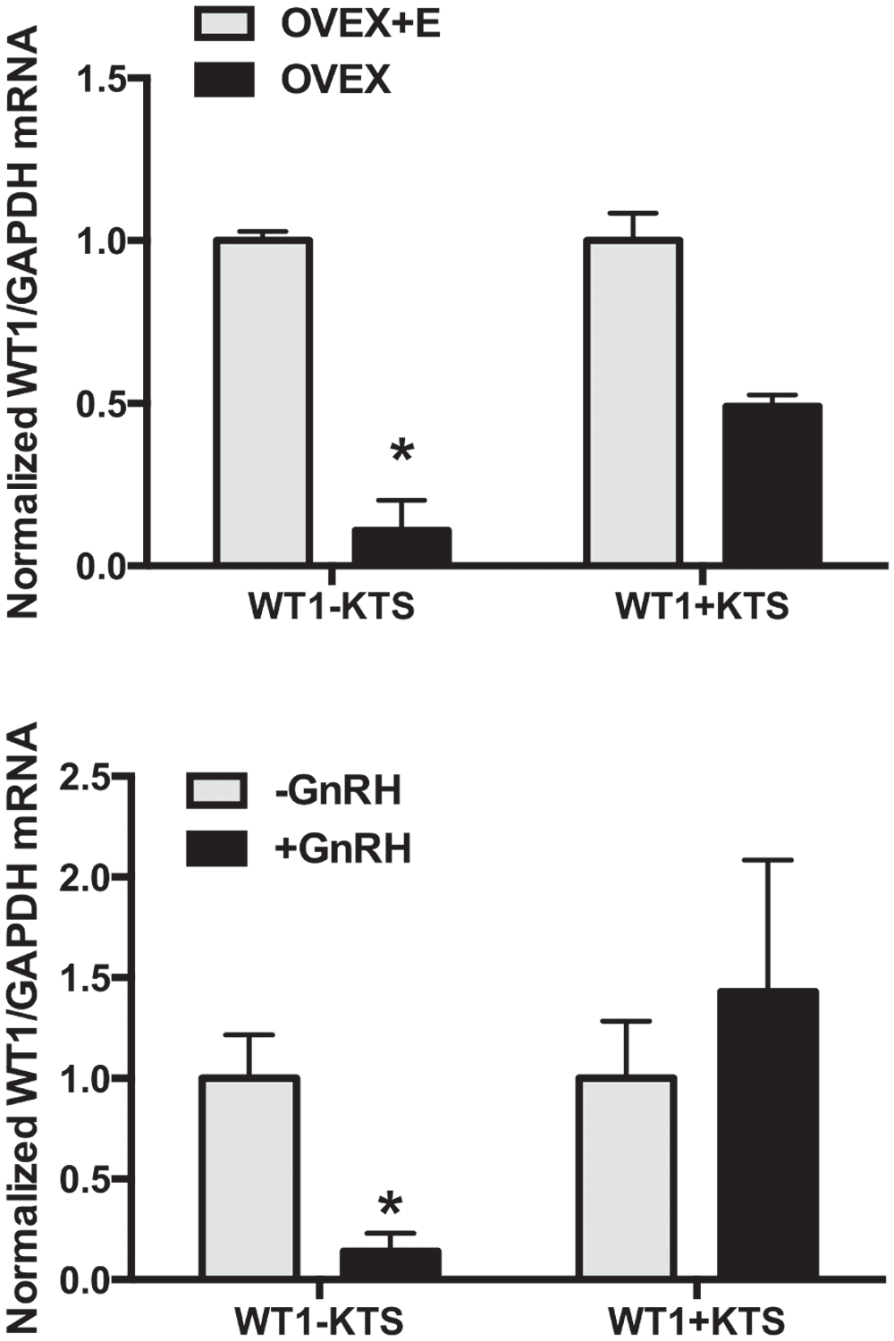
Expression and regulation of WT1 variant mRNA in mouse pituitary cells. WT1 variant mRNAs were measured by quantitative RT-PCR and normalized to GAPDH mRNA in the same samples in pituitary glands from: (Upper panel) Ovariectomized (OVEX) mice treated *in vivo* with vehicle or E2 for 3 d or (Lower panel) Pituitary glands from intact mice treated in culture with 5 nM GnRH for 30 min. Data are from 4 independent determinations and expressed as the mean ± SEM. * P < 0.05 OVEX vs OVEX + E, or Control vs GnRH.

To test if WT1 variants are expressed and regulated in normal gonadotrope cells in addition to the clonal mouse gonadotrope cell line, mRNA was collected from mouse pituitary cells under different physiological conditions and WT1 mRNA variants were measured. As shown in Fig. 9, both WT1 variants were expressed in pituitary glands from ovariectomized female mice (upper panel). WT1(-KTS) expression was highest in ovariectomized mice treated with 17β-estradiol compared to those treated with vehicle alone. WT1(-KTS) mRNA was not significantly changed between the two groups, although the trend was to lower expression in pituitary cells from ovariectomized mice. Interestingly, lower expression of WT1(-KTS) in ovariectomized females versus treatment of ovariecomized mice with E2 correlates with more robust GnRH pulse patterns noted in ovariectomized animals [3]. In mouse pituitary cells treated in culture with GnRH (lower panel), WT1(-KTS) mRNA levels were decreased approximately 75% by GnRH, while WT1(+KTS) mRNA was not significantly changed with GnRH treatment.

## Discussion

In this work, we show for the first time that WT1 regulates LHβ transcription, and that the –KTS and +KTS have differential roles in this regulation. The function of WT1 is well established in the urino-genital (kidney and gonads) system [18, 19, 20, 21], but its role has been largely unexplored in the pituitary. We demonstrate that WT1 is expressed in LβT2 cells, an immortalized mouse gonadotrope cell line, is regulated by GnRH (Fig. 1 and Fig. 2), binds to the LHβ promoter, and regulates promoter activity. Both WT1 mRNA variants are expressed in normal mouse pituitary (Fig. 9) and the WT(-KTS) isoform also appears to be significantly regulated by GnRH.

As a Zinc-finger transcription factor, WT1 binds to GC-rich regions common to Egr1 and SP1, but often under different physiological conditions or with different biological outcomes [21, 22, 23, 37]. For example, WT1 and Egr1 have been shown to regulate the expression of the STIM1 gene by competing for one of the common binding sites on the STIM1 promoter. Egr1 stimulates STIM1 expression and WT1 antagonizes this effect by binding to Egr1 binding sites [37]. In addition, co-ordination of the differential binding pattern of the transcription factors WT1, Egr1, and Sp1 in a time-dependent manner was shown to be involved in the regulation of the α Isoform of the human thromboxane A2 receptor during megakaryocyte differentiation [23]. In response to PMA, there is an initial induction of Egr1 and reduction of WT1 association with this promoter, followed by SP1 occupancy after sustained PMA treatment [23].

Our data demonstrates mRNA expression of the two most prevalent WT1 splice variants, –KTS and +KTS, and presence of the WT1 protein in untreated cells (Fig. 1). Chromatin immunoprecipitation (ChIP) assays showed the association of WT1 to LHβ promoter in untreated cells, suggesting that WT1 could modulate basal LHβ expression (Fig. 1). The differential regulation of WT1 and Egr1 by GnRH in LβT2 cells (Fig. 2), suggests that Egr1 and WT1 may play somewhat different roles in LHβ expression. WT1, but not Egr1, was found to be expressed in untreated cells, and while Egr1 protein is dramatically induced by GnRH, WT1 protein is reduced by approximately 50%. GnRH stimulates LHβ transcription, and induces cyclic binding of the transcription factors Egr1 and SF1, and RNAPol II, on the LHβ promoter to drive transcription [10]. In this work, we showed that GnRH stimulates the cyclic association of both WT1 and Egr1 to the LHβ promoter, but Egr1 occupancy lags that of WT1 as GnRH must first stimulate Egr1 protein synthesis (Fig. 3). These data suggest involvement of WT1 in GnRH-stimulated LHβ transcription as well. There is currently no antibody that distinguishes between the WT1(-KTS) and WT1(+KTS) variants, and we cannot distinguish if both variants are binding to the promoter, or if one variant binds preferentially under basal or GnRH-stimulated conditions, by this method.

When we performed siRNA knock-down studies, knock-down of WT1(-KTS) mRNA alone did not significantly alter basal LHβ mRNA transcription, measured by levels of LHβ primary transcript mRNA, but decreased GnRH-stimulated transcription by approximately 50%, correlating with decreases in Egr1 mRNA primary transcript under the same conditions (Fig. 4A). In comparison, overexpression of WT1(-KTS) significantly increased GnRH stimulation of LHβ promoter activity by approximately 3-to 4-fold at the highest concentrations (Fig. 5); a small but significant increase in basal expression was also noted with the smaller promoter construct containing Egr1 sites and the proximal GnRH response region. Based on mutation data (Fig. 7) only the 3’-Egr1 site, and not SF1 sites, is required for WT1(-KTS) activity on LHβ. This is in keeping with a positive role for WT1(-KTS) in LHβ transcription, via Egr1 expression and Egr1 binding sites. The Egr1 promoter contains GC-rich motifs that bind Egr1 [38] and potentially other related transcription factors such as WT1, so WT1(-KTS) regulation of this gene may be direct.

In contrast, when both mRNA isoforms for WT1 were decreased, and endogenous WT1 protein decreased by >95% (Fig. 4B), basal LHβ transcription was significantly increased (approximately 2-fold), and GnRH treatment decreased transcription below basal levels (Fig. 4B). GnRH-stimulated Egr1 primary transcript mRNA was not reduced, as was the case when only WT1(-KTS) was knocked down. WT1(+KTS) may antagonize the effects of WT1(-KTS) on Egr1 expression directly, or act via other genes and pathways. Overexpression of WT1(+KTS) alone decreased both basal and GnRH-stimulated LHβ promoter activity (Fig. 6) and required both the 3’-Egr1 site and the SF1 sites in the LHβ promoter (Fig. 8).

**Fig 10:**
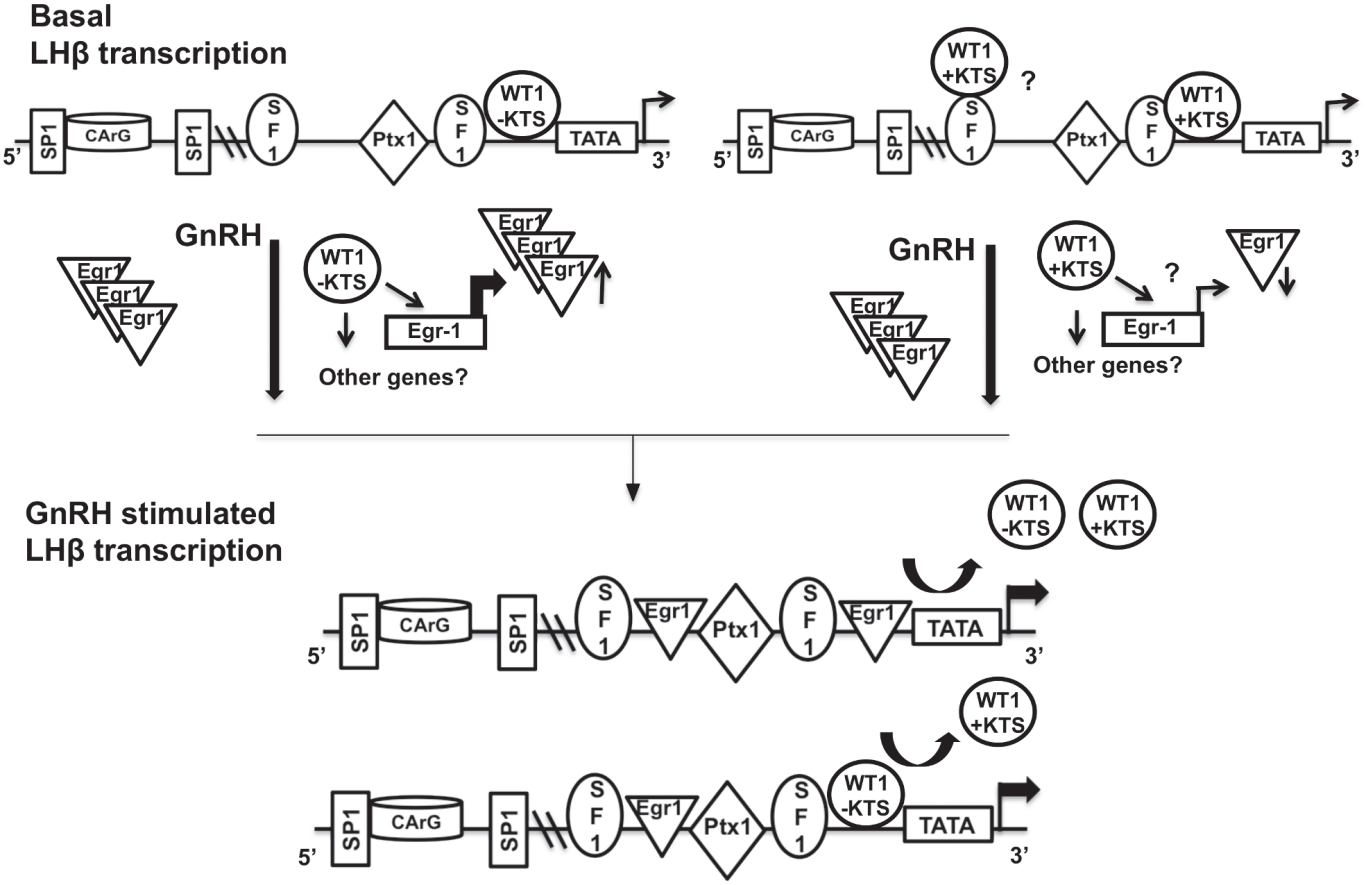
Model of WT1 variant actions on the LHβ promoter. WT1 (-KTS) may associate directly with the 3’Egr1 site of the LHβ promoter to stimulate basal promoter activity, but in the presence of GnRH stimulates Egr1 expression. Egr1 then binds to both the 3’- and 5’-Egr1 sites on the promoter and further increases LHβ transcription. WT1 (+KTS) may associate with the 3’-Egr1 site, but also requires the SF1 site for biological activity. Decreased expression of WT1 (+KTS) would stimulate basal LHβ expression as the suppressor is reduced. The isoforms likely compete for association to the LHβ promoter at the same sites.

These data suggest a scenario in which WT1(+KTS) acts to suppress LHβ, while WT1(-KTS) acts primarily to enhance GnRH stimulation of LHβ promoter activity (Fig. 10). WT1 binding to the LHβ promoter under basal (no GnRH) conditions could help maintain low promoter activity, and WT1 is associated with the promoter in the absence of GnRH (Fig. 1). WT1(-KTS) would have positive effects on the promoter, while WT1(+KTS) would be suppressive, and there may be competition between the two isoforms for association with the LHβ promoter at the same gene site. Because WT1 exerts its actions only via the 3’-Egr1 site, stimulated Egr1 expression in the presence of GnRH would presumably result in more effective promoter occupancy at both Egr1 sites, and greater LHβ transcription. This is consistent with the lesser ability of WT1(-KTS) alone to increase LHβ promoter activity compared to promoter stimulation with GnRH (Fig. 5), and the ability of Egr1 overexpression to effectively substitute for GnRH in stimulating LHβ promoter activity [10, 32]. Interestingly, WT1(-KTS) appears to be more tightly regulated in normal pituitary cells (Fig. 9). GnRH treatment would decrease expression of the repressor WT1(+KTS) and the stimulator WT1(-KTS), but because Egr1 is more effective on LHβ than WT1(-KTS), overall LHβ promoter activity will be much higher.

The failure of GnRH to stimulate LHβ with complete WT1 protein knock-down in Fig. 4 is less straightforward. Increased basal activity may occur due to loss of suppression by WT1(+KTS), but changes in Egr1 are not sufficient to explain this result and both WT1 isoforms may regulate other genes and pathways that could influence LHβ transcription. The +KTS variant was shown to be involved in RNA processing and RNA metabolism [44], but a recent report showed that +KTS can also bind to DNA on the planar cell polarity gene promoter SCRIBBLE and regulate its transcription in developing kidney [45]. WT1 acts synergistically with the transcription factor SF1 to regulate the expression of the Mullerian inhibiting substance gene during development of male gonads [18], and to regulate the expression of α-inhibin in Sertoli cells [39]. Both DNA binding and protein-protein interactions with SF1 may play a role in LHβ promoter regulation by WT1(+KTS), and additional protein-protein interactions could also contribute to these biological effects.

Differential regulation of transcription by the two WT1 splice variants has been noted for some other genes. For example, WT1+KTS strongly represses the insulin receptor promoter, whereas repression by WT1–KTS is more moderate and occurs only in the presence of additional C/EBPβ or a dominant negative p53 [30]. The –KTS splice variant of WT1 has been shown to stimulate α-inhibin expression in Sertoli cells of the testis but the +KTS variant had no such effect [39]. For LHβ, the two variants appear to have opposing roles in transcription, and the relative balance between the two forms may be crucial. Interestingly, mutations in intron 9 of the WT1 gene, where alternative splicing to generate WT1+KTS and –KTS occurs, result in Frasier syndrome, including sex reversal and developmental defects in kidney and gonads [40, 41, 42, 43]. Mutations in some Frasier syndrome patients result in the predicted decrease of the WT1(+KTS) isoform and diminution of the WT1 (+KTS/-KTS) isoform ratio [40, 41]. Given the crucial role of WT1 in development of the reproductive organs and urogenital tract, and the necessary feedback between steroids on the hypothalamus and pituitary, studies to evaluate a potential role of *WT1* mutations on pituitary function are difficult. However, a *WT1* mutation (IVS9+5G>A) that causes Frasier syndrome has also been linked to hypergonadotropic hypogonadism and increased serum levels of gonadotropins (LH and FSH) in patients [42]. In at least one patient with a *WT1* mutation (IVS9+4C>T) and high basal LH, both a decrease in the WT1(+KTS) and an increase in the WT1(–KTS) isoforms was observed [43]. These observations are in agreement with the increase in basal LH transcription observed in our siRNA studies when knock-down of both WT1 +KTS and –KTS WT1 occurred, and not when only the WT1(-KTS) variant was reduced.

Overall our data indicate that the WT1(+KTS) and (-KTS)splice variants play a differential and opposing role in regulating LHβ transcription. The role of WT1 as an activator or repressor seems to be context and promoter specific, and could also be influenced by the ratio of its splice variants if they exert opposing effects on the same promoter. The proximal GnRH responsive element region containing the two Egr-1 binding sites, and the two SF1 binding sites, is sufficient to exert the effects of WT1, and this region of the promoter is identical in rodents and humans [37]. Both direct WT1 binding to DNA and protein-protein association with SF1 play a role in WT1 function in this system. The two splice variants (-KTS and +KTS) of WT1 acting as a positive and negative regulator, respectively, to regulate LHβ gene transcription, defines a novel regulatory role of WT1 in pituitary gonadotropes and the reproductive system.
